# A randomized controlled trial of brief Somatic Experiencing for chronic low back pain and comorbid post-traumatic stress disorder symptoms

**DOI:** 10.1080/20008198.2017.1331108

**Published:** 2017-05-30

**Authors:** Tonny Elmose Andersen, Yael Lahav, Hanne Ellegaard, Claus Manniche

**Affiliations:** ^a^ Department of Psychology, University of Southern Denmark, Odense, Denmark; ^b^ Center of Excellence for Mass Trauma, Tel Aviv University, Tel Aviv, Israel; ^c^ Spine Center and University of Southern Denmark, Middelfart, Denmark

**Keywords:** Post-traumatic stress, Somatic Experiencing, pain, low back pain, RCT

## Abstract

**Background:** It is well documented that comorbid post-traumatic stress disorder (PTSD) in chronic pain is associated with a more severe symptom profile with respect to pain, disability and psychological distress. However, very few intervention studies exist targeting both PTSD and pain. The current study is the first randomized controlled trial evaluating the effect of the body-oriented trauma approach of Somatic Experiencing (SE) for comorbid PTSD and low back pain. Although the method is well recognized by clinicians and widely used, SE still needs to be tested in a randomized clinical trial in comparison with an active control group.

**Objective:** The aim of the current study was to compare the effect of an SE intervention in addition to treatment-as-usual (TAU) for patients with chronic low back pain and comorbid PTSD compared to TAU alone.

**Method:** The study was a two-group randomized controlled clinical trial. A cohort of patients (*n* = 1045) referred to a large Danish spine centre between February 2013 and October 2014 were screened for PTSD and randomized to either TAU (4–12 sessions of supervised exercises for low back pain) or TAU plus SE (6–12 sessions). In total, 91 patients fulfilled the inclusion criteria and volunteered to participate in the study. Treatment effects were evaluated by self-report questionnaires comparing baseline measures with 12-month follow-up measures.

**Results:** The additional SE intervention significantly reduced the number of PTSD symptoms compared with TAU alone, corresponding to a large effect size. Also, fear of movement was significantly reduced (moderate effect size). Both groups achieved a large reduction in pain-catastrophizing, disability and pain.

**Conclusions:** A brief additional SE intervention was found to have a significant effect on PTSD and fear of movement compared to TAU alone. However, the overall effect of SE was less than expected and the clinical importance of the effects can be questioned.

Low back pain is a very common health problem and rated among the top 10 global diseases that account for most years lived with disability (Vos et al., 2012). Also, psychological comorbidities such as anxiety and mood disorders are very common in chronic low back pain (Von Korff et al., 2005). It is well documented that comorbid post-traumatic stress disorder (PTSD) in chronic pain is associated with a more severe symptom profile with respect to pain, disability and psychological distress (Andersen, Andersen, & Andersen, 2014; Andersen, Elklit, & Vase, 2011; DeCarvalho, 2010; Moeller-Bertram, Keltner, & Strigo, 2012; Otis, Keane, Kerns, Monson, & Scioli, 2009). Although promising models and mechanisms have been suggested explaining the co-existence of PTSD and pain, only a small number of intervention studies exist targeting both PTSD and pain. Unfortunately, most studies have been case studies or limited by a lack of randomization. To our knowledge, only two randomized controlled trials (RCT) exist targeting comorbid PTSD and pain. Both are cognitive behavioural therapy (CBT) trials for motor vehicle-related PTSD. Beck, Coffey, Foy, Keane, and Blanchard (2009) compared group CBT with a minimal contact comparison condition (*n* = 44). The CBT intervention was associated with a moderate reduction in PTSD symptoms. However, change in pain severity was not different from the controls. The second trial was a pilot study (*n* = 26) on whiplash-associated disorders assessing the effect of trauma-focused CBT compared to a waiting list. A moderate reduction in neck-related pain and disability as well as PTSD was found compared to the waiting list (Dunne, Kenardy, & Sterling, 2012). However, the results should be interpreted with caution, since both studies were under-powered and without an active control condition. Moreover, only change in PTSD symptoms was considered a clinically important effect, corresponding to a large effect size (Dunne et al., 2012). Although statistically significant, pain and disability scores did not reach the predetermined cut-off for clinically important difference (CID).

While exposure to trauma memories is one of the most common approaches to treating PTSD (Peri, Gofman, Tal, & Tuval-Mashiach, 2015), the so-called ‘active ingredients’ across treatments are still being debated (see Schnyder et al., 2015). Recently, it has been suggested that more subtle processes related to the therapeutic relationship and embodied cognition may be common underlying mechanisms associated with emotion regulation and fear extinction across therapeutic approaches (Peri et al., 2015). Also, a number of patients have difficulties tolerating exposure in vivo or to trauma memories resulting in elevated arousal and the risk of dropping out of treatment (Wald & Taylor, 2008). Moreover, trauma-related memories are not necessarily stored in a coherent sequential timeline, making retrieval of memories difficult (Van der Kolk & Fisler, 1995). Finally, in more complex traumas, dissociation may interfere with the processing of trauma memories, causing exposure therapy to be less than optimal (Resick, Suvak, Johnides, Mitchell, & Iverson, 2012). For these reasons, alternative or additional methods are needed to improve treatment tolerability and processing of maladaptive procedural memories. Such a method may be found in a more bodily-oriented intervention such as Somatic Experiencing (SE).

SE is a relatively new trauma therapy developed by Levine (1977, 1997, 2010). SE differs from traditional CBT and exposure-based interventions in its bodily focus on interoception and musculoskeletal sensations rather than cognitions and emotional experiences. SE also includes techniques known from interoceptive exposure for panic attacks, by combining arousal reduction strategies with mild exposure therapy (Barlow & Craske, 2007). Unlike conventional psychotherapy, which focuses largely on verbal cognitive processes, traumatic memories are targeted indirectly by gradually guiding the patients to develop an increasing tolerance for difficult bodily sensations and emotions. In SE, the rationale is somewhat similar to that of mindfulness, that sustained attention to interoceptive sensations is a means to stay mindful in the present moment and thereby facilitate new interoceptive experiences that contradict those of overwhelming anxiety and helplessness associated with the trauma (Payne, Levine, & Crane-Godreau, 2015). For instance, in traffic accidents, where pain is part of the trauma, sensitization to pain may develop because pain serves as a reminder of the traumatic event and fear of pain may develop as a consequence (Sharp & Harvey, 2001). Experimental studies have found that interoceptive training is associated with reduced activation in neural networks linked to involuntary cognitive elaboration, and increased recruitment of viscero-somatic neural regions linked to momentary awareness of internal sensation (Farb et al., 2007). Finally, disrupting automatic cognitive thoughts by interoceptive attention is found to lower depression and improve access to bodily sensations during sadness (Farb et al., 2007). In addition, a growing number of studies have found acceptance of bodily sensations to be an important mechanism in coping with pain and distress (McCracken & Morley, 2014; Veehof, Oskam, Schreurs, & Bohlmeijer, 2011). Few case studies have focused on interoceptive techniques in the treatment of comorbid pain and PTSD (Shipherd, 2006; Wald & Taylor, 2006; Wald, Taylor, Chiri, & Sica, 2010). These results indicate that an interoceptive approach may have an effect on trauma symptoms, distress and somatic symptoms. In the only existing non-randomized controlled study of SE, Leitch, Vanslyke, and Allen (2009) found that a brief SE intervention was able to reduce PTSD symptoms and psychological distress in social service workers following the hurricanes Katrina and Rita compared to controls. No significant reduction in somatization was found.

Theoretically, SE may target important mechanisms as described in the mutual maintenance model (Sharp & Harvey, 2001) and the shared vulnerability model (Asmundson & Katz, 2009). In the models, it is outlined how elevated levels of arousal, attention bias, anxiety sensitivity, catastrophic thinking and avoidance behaviours are mechanisms maintaining both PTSD and pain. While PTSD and pain are thought to be mutually maintaining conditions in the first six months after a trauma, in the more chronic phase it has been found that only PTSD was maintaining pain (Jenewein, Wittmann, Moergeli, Creutzig, & Schnyder, 2009; Stratton et al., 2014). For these reasons, targeting comorbid PTSD in pain management seems promising. Given the limited research on interventions for comorbid PTSD in chronic low back pain, this study addresses a gap in knowledge by reporting the results of the first RCT evaluating the effect of SE for comorbid PTSD and chronic low back pain.

The aim of the current study was to test whether an additional trauma-focused intervention (SE) targeting PTSD related to unresolved trauma in combination with supervised exercises for low back pain (treatment-as-usual) would reduce pain-related disability as suggested by the mutual maintenance model (Sharp & Harvey, 2001). First, it was hypothesized that the additional SE intervention would reduce pain-related disability compared to TAU alone. Secondly, compared to TAU alone, it was hypothesized that the additional SE intervention would reduce all secondary outcomes: pain, PTSD, catastrophic thinking about pain and fear of movement.

## 1. Methods

### 1.1. Study design and participants

The study is a two-group randomized controlled clinical trial in which participants (*n* = 91) were recruited consecutively from a large Danish spine centre in the Region of Southern Denmark, between February 2013 and October 2014 with follow-up at 12 months. Ethics approval was obtained from the local science ethics committee (trial number S-20120154) and all participants gave written informed consent before study entry. Patients were recruited as part of the standard screening procedure at the spine centre. The centre is a government-funded facility where patients can be referred from anywhere within a catchment area of 1.2 million people. Department personnel perform multidisciplinary assessments of patients with spinal pain after referral from general practitioners. A standardized clinical examination and use of magnetic resonance imaging (MRI) are central elements. The clinical examination included spine movement tests, Lasegue’s test, absence of patellar and Achilles reflexes, etc. No psychological tests were applied. If the patients evaluate that their improvement in low back pain has not been satisfactory with primary care treatment after 1–2 months they have a right to be referred to the spine centre.

Patients were considered eligible for inclusion if they met the criteria for possible sub-clinical or clinical PTSD (see Procedures below) as measured on the Harvard Trauma Questionnaire part IV (Mollica et al., 1992), were between 18–65 years of age, and proficient in written and spoken Danish. Exclusion criteria were known serious psychiatric comorbid diseases such as bipolar, depression, psychosis or drug dependence. Other ongoing psychotherapeutic interventions also led to exclusion.

### 1.2. Randomization and masking

Patients were randomized by random permuted blocks of six by the study statistician at the spine centre. Randomization was consecutively numbered in sealed opaque envelopes. Patients were randomly allocated to one of the two conditions: TAU or TAU + SE. Treatment was initiated within two weeks after randomization. Measurements of effect were carried out at baseline before randomization and after treatment (12 months post-randomization). Clinicians were not blinded to which interventions the patients received, however, the study statistician who conducted the analysis was blinded to treatment allocation.

### 1.3. Procedures

All participants received TAU. This treatment consisted of supervised exercises for low back pain delivered in 4–12 sessions and performed by physiotherapists in the centre or in primary sector clinics according to the European guidelines for the management of chronic low back pain (Airaksinen et al., 2006).

### 1.4. The Somatic Experiencing intervention

In the SE intervention, patients received 6–12 hours of SE therapy delivered by a certified SE therapist who was also a pain nurse with several years of experience. The SE intervention followed the nine-step model as outlined by Peter Levine (2010) and involved gradually eliciting awareness of body sensations associated with the traumatic event. By the process of ‘titration’, patients were gradually encouraged to access somatic activation, feelings and body sensations as means to restore equilibrium to the autonomic nervous system and thereby alleviate hyperarousal, re-experiencing and avoidance of trauma-related experiences and thoughts.

### 1.5. Outcome measures

All outcomes were obtained by an investigator who was blinded to group allocation at baseline and at follow-up 12-months post-randomization. Demographic characteristics such as age, gender, onset of pain, and years since traumatic events were obtained at baseline. The outcomes were as follows:

### 1.6. Primary outcome

Disability was measured with Roland Morris Disability Questionnaire (RMDQ; Roland & Morris, 1983). The RMDQ is a self-reported outcome measuring the level of disability related to low back pain. The level of disability was measured on 23 statements covering six different domains: physical ability/activity, sleep/rest, psychosocial level of functioning, household management, eating, and pain frequency. Each statement was scored 1 if the patient felt that the statement was descriptive of their circumstances and scored 0 if not. The total RMDQ score ranges from 0 (no disability) to 23 (maximal disability). Scores were converted to percentages with 24 corresponding to 100% disability. Both internal consistency (Cronbach’s alpha α = 0.84–0.96) and test-retest reliability (*r* = 0.83–0.91) of the RMDQ are good (Smeets, Köke, Lin, Ferreira, & Demoulin, 2011). In the current study, internal consistency measured by Cronbach’s alpha was α = .88.

### 1.7. Secondary outcomes

Pain intensity was measured as the average score of three 11-point Likert scales measuring peak pain intensity, average pain intensity over the past two weeks as well as current pain intensity (Manniche, Asmussen, Lauritsen, Vinterberg, & Kreiner, 1994). Each scale measured pain intensity on a 0–10 numerical rating scale (NRS; Jensen, Karoly, & Braver, 1986) with 0 defined as no pain and 10 as the worst imaginable pain. Internal consistency measured by Cronbach’s alpha was α = .86.

PTSD symptoms were measured using the Harvard Trauma Questionnaire part IV (Mollica et al., 1992). The Harvard Trauma Questionnaire consists of 17 items with a 4-point Likert scale (1 = not at all to 4 = very often). The 17 items relate to PTSD’s core clusters within the Diagnostic and Statistical Manual of Mental Disorders (APA, 1994), 4th Edition (DSM-IV): avoidance (7 items), re-experiencing (5 items), and hyperarousal (5 items). An item was deemed to be positively endorsed if scores were ≥ 3. The Harvard Trauma Questionnaire follows the diagnostic criteria for the PTSD diagnosis according to the DSM-IV. The scale thus makes it possible to measure both the severity of symptoms and to estimate the prevalence of possible PTSD. Following the DSM-IV, a possible PTSD diagnosis was proposed if participants reported at least one re-experiencing symptom, three avoidance symptoms, and two hyperarousal symptoms. Possible sub-clinical PTSD was proposed in cases where the patients either missed one symptom of avoidance or hyperarousal. The Harvard Trauma Questionnaire self-report measure of PTSD has previously been reported as having an 88% concordance with interview-based estimates of PTSD (Mollica et al., 1992). The internal consistency, measured by Cronbach’s alpha, was α = .82. Preceding the completion of the Harvard Trauma Questionnaire, the patients were asked to identify significant traumatic stressors from a list that included experiences of both direct and indirect exposure to traumatic events. Moreover, the participants were asked to report which event they experienced as the primary traumatic event (index trauma). The items were based on a variety of experiences included in the diagnostic criteria for traumatic exposures, according to the DSM-IV.

The Pain Catastrophizing Scale (Sullivan, Bishop, & Pivik, 1995) was used to measure catastrophic thinking related to pain. Its instructions ask participants to reflect on past painful experiences, and to indicate the degree to which they experienced each of 13 thoughts or feelings when experiencing pain, on a 5-point Likert scale (0 = not at all, 4 = all the time). A scale sum score was calculated from all items, with a high score indicating a high level of pain catastrophizing. Internal consistency measured by Cronbach’s alpha was α = .91.

Fear of re-injury due to movement was measured with the Tampa Scale for Kinesiophobia (TSK; Kori, Miller, & Todd, 1990). TSK is a 17-item scale assessing fear of movement on a 4-point Likert scale ranging from 17 to 68 with higher scores indicating higher levels of kinesiophobia. The scale is commonly used in diverse chronic pain samples and has good construct and predictive validity (Vlaeyen, Kole-Snijders, Boeren, & van Eek, 1995). Internal consistency measured by Cronbach’s alpha was α = .81.

### 1.8. Statistics

The sample size of 90 patients was calculated a priori. This sample size provided 80% power to detect a moderate effects size for the primary outcome measures. This calculation assumed an α of 0.05 and allowed for up to 10% loss to follow up and non-compliance.

Results are presented as mean and standard deviation (*SD*) in the text and mean and standard error of the mean (SEM) in figures. None of the measured variables deviated from normality (Kolmogorov-Smirnov test: *p *> .05) and parametric statistics were used for analysis. Baseline characteristics were analysed with chi square tests for dichotomous data and with independent sample *t*-tests for continuous data. Pearson’s correlations were used to assess correlations for all outcomes at baseline.

Two-way repeated measures analysis of variance (ANOVA) were used for analysis of the outcome measures with the factor *time* (baseline and 12-month follow-up) as a repeated measure and *group* (SE and SE + TAU) as the between-subjects factor. Effect sizes of the difference in outcome measures between groups were calculated based on partial eta squared (for a small effect η^2^p = .02, medium effect η^2^p = .13 and large effect η^2^p = .26). Cohen’s *d* was reported for significant within-group effects.

Missing data is very common in longitudinal designs (Collins, Schafer, & Kam, 2001). In order to handle missing data and to create a reliable dataset, participants were only included if they participated in both waves of measurement (T1–T2). Overall 0–30.6% of data were missing across waves. To decide whether the data had missing values in a pattern that was random, we conducted analyses of differences between these groups in all of the variables, using Little’s Missing Completely at Random (MCAR) test (Collins et al., 2001). The analysis revealed that the data were missing completely at random, chi square (720) = 698.94, *p *= .706. Although the mechanism of missing data was proven to be missing at random and not related to the observed data, we decided to use the more advanced method of maximum likelihood using SPSS 22. As the current data were longitudinal, the maximum likelihood method was considered to be the optimal method for attrition of participants over time (Collins et al., 2001). This method is optimal in order to avoid biased data (Schafer & Graham, 2002), as compared to conventional methods such as arithmetic mean, listwise or pairwise deletion.

## 2. Results

### 2.1. Baseline characteristics

**Figure 1.**
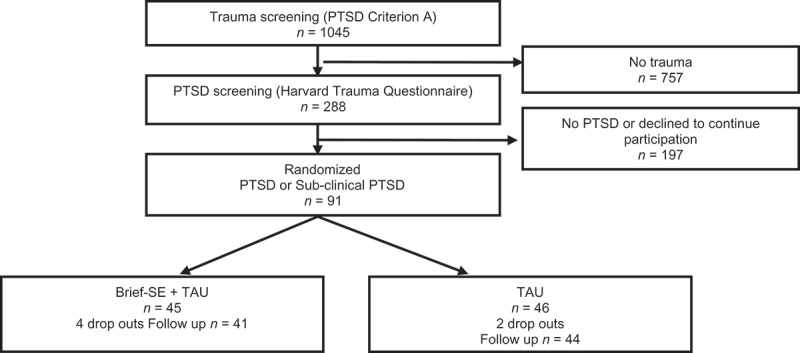
Trial profile.

Figure 1 illustrates the patient flow in this study. Of the 1045 eligible patients, 288 had experienced a traumatic event (DSM-IV criteria A). Patients were screened for possible PTSD/sub-clinical PTSD. In total, 91 patients fulfilled the inclusion criteria and volunteered to participate in the study (*n* = 45 for SE group, *n* = 46 for controls).

The mean age was 50.6 years (*SD* = 9.31) and 54.2% were females. The mean years since the traumatic event(s) was 10.6 (*SD* = 9.18). The mean number of traumatic events experienced by each participant was 1.36 (*SD* = 0.75). The traumatic events reported were as follows: accidents (33%), assaults (sexual or violent; 27%), serious illness or disease of oneself or a close relative (22%), death of a close relative (32%) and other (18%).

Compared at baseline, there were no significant differences in primary or secondary outcomes between the two groups with the exception of gender. There were more females in the SE group (65.9%) compared with the TAU group (42.9%) χ^2^(1) = 4.42, *p* = .048.

Significant positive correlations were found between PTSD and all outcomes with the exception of kinesiophobia. Correlations between all outcomes are presented in Table 1.

**Table 1.** Baseline correlations for study measures.

Measure	1	2	3	4
1. PTSD	–			
2. PCS	.29**	–		
3. TSK	.13	.48***	–	
4. Pain	.28**	.33**	.25*	–
5. RMDQ	.41***	.43***	.36**	.46***

PTSD = number of symptoms above an item cutoff score ≥ 3; PCS = Pain Catastrophizing Scale; TSK = Tampa Scale for Kinesiophobia; Pain = average score of three 11-point Likert scales; RMDQ = Roland Morris Disability Questionnaire 0–100% disability.

*p < .05. **p < .01. ***p < .001.

### 2.2. Change in primary and secondary outcomes

In order to compare the change scores over time between the two groups, we conducted a series of mixed analyses of variance with study group as a between-subjects variable; time of measurement as within-subject variable; and severity of primary and secondary outcomes as dependent variables. Because these analyses were interdependent, we controlled for Type I error with Bonferroni corrections for multiple comparisons. Relevant means and standard deviations are presented in Table 2.

**Table 2.** Means and standard deviations of outcomes at baseline and 12 months among SE group and control group.

	SE group	Control group
	*M* (*SD*)	*M* (*SD*)
RMDQ*		
Baseline	68.14 (22.22)	67.47 (20.95)
12-months	58.98 (26.10)	59.10 (27.99)
Change	9.2%	8.4%
Pain		
Baseline	6.42 (2.23)	6.22 (1.78)
12-months	5.25 (2.36)	5.49 (2.41)
Change	11.7%	7.3%
PTSD		
Baseline	10.70 (3.30)	10.00 (3.00)
12-months	8.68 (5.30)	10.33 (4.75)
Change	11.9%	−1.9%
PCS		
Baseline	25.66 (9.85)	24.98 (9.90)
12-months	22.29 (10.37)	23.97 (11.11)
Change	6.5%	1.9%
TSK		
Baseline	44.51 (5.72)	45.08 (5.87)
12-months	41.98 (5.64)	44.73 (6.09)
Change	5.0%	0,7%

* = Primary outcome; RMDQ = Roland Morris Disability Questionnaire 0–100% disability; Pain = average score of three 11-point Likert scales; PTSD = number of symptoms above an item cutoff score ≥ 3; PCS = Pain Catastrophizing Scale; TSK = Tampa Scale for Kinesiophobia.

### 2.3. Change in primary outcome

The ANOVA conducted on RMDQ revealed non-significant effects for grouping, *F*(1, 83) = .01, *p = *.96, η^2^p = .00 as well as for time x group interaction, *F*(1, 83) = .04, *p = *.84, η^2^p = .00. The effect for time was significant, *F*(1, 83) = 19.68, *p < *.001, η^2^p = .19. Both study groups showed a decrease in RMDQ levels from baseline to post treatment.

### 2.4. Change in secondary outcomes

The ANOVA conducted on pain revealed non-significant effects for grouping, *F*(1, 83) = .00, *p = *.98, η^2^p = .00, and for time x group interaction, *F*(1, 83) = 1.29, *p = *.26, η^2^p = .02. The effect for time was significant, *F*(1, 83) = 23.62, *p < *.001, η^2^p = .22. Both study groups showed a decrease in pain levels from baseline to post treatment.

**Figure 2.**
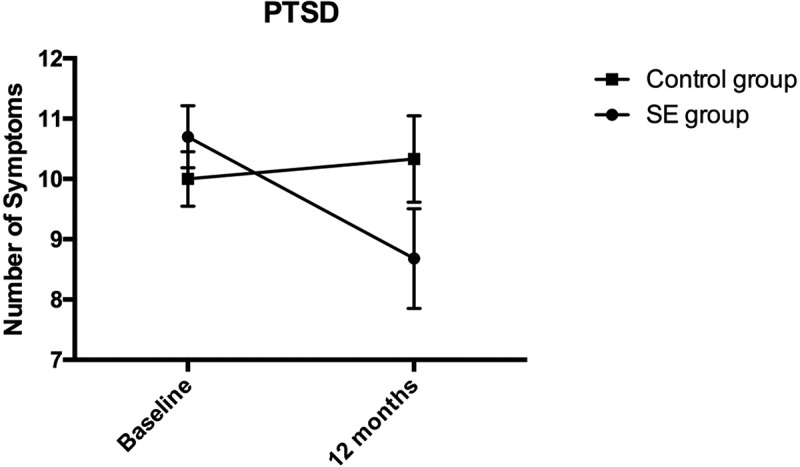
Number of PTSD symptoms.

The ANOVA conducted on PTSD revealed non-significant effects for grouping, *F*(1, 83) = .39, *p *= .53, η^2^p = .01, and for time, *F*(1, 83) = 2.76, *p *= .10, η^2^p = .03. However, the time x group interaction was significant, *F*(1, 83) = 5.28, *p *= .02, η^2^p = .06. Simple effects revealed that while the effect of time on severity of PTSD symptoms was non-significant among controls (Mean Difference = .33, *p = *.65), it was significant among the SE group indicating a decrease in PTSD symptoms from pretreatment to post-treatment (Mean Difference = −2.03, *p < *.01, Cohen´s *d = *0.46). The group difference for PTSD symptoms is illustrated in Figure 2.

The ANOVA conducted on Pain Catastrophizing (PCS) revealed non-significant effects for grouping, *F*(1, 83) = .06, *p *= .80, η^2^p = .00, as well as for time x group interaction, *F*(1, 83) = 1.45, *p *= .23, η^2^p = .02. The effect of time was significant, *F*(1, 83) = 4.95, *p *= .03, η^2^p = .06. Both study groups showed a decrease in Pain Catastrophizing levels from baseline to post treatment.

**Figure 3.**
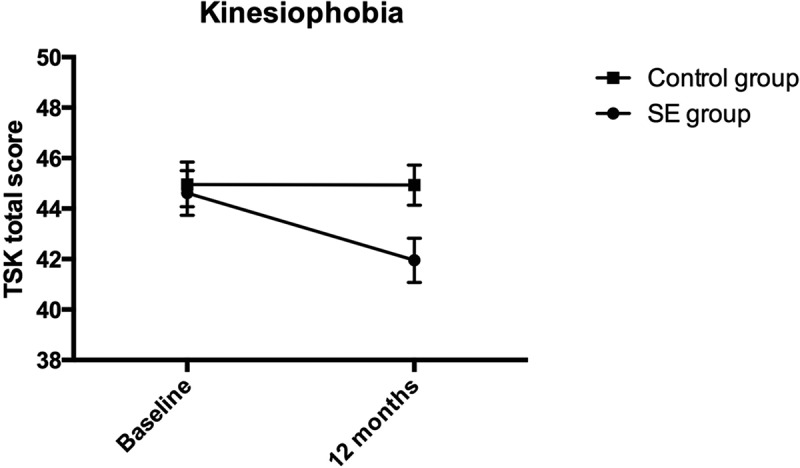
Kinesiophobia.

The ANOVA conducted on TSK revealed non-significant effects for grouping, *F*(1, 83) = 2.16, *p *= .15, η^2^p = .03. The analysis revealed significant effects for time, *F*(1, 83) = 6.27, *p = *.01, η^2^p = .07. Although the effect for time x group interaction was non-significant, *F*(1, 83) = 3.63, *p *= .06, η^2^p = .04, simple effects revealed that while among controls the change with time was non-significant (Mean Difference = .35, *p = *.67) among the SE group, there was a significant change from baseline to post treatment (Mean Difference = 2.53, *p = *.003, Cohen´s *d = *0.45). The group difference is illustrated in Figure 3.

## 3. Discussion

The aim of the current study was to compare the effect of a brief SE intervention in addition to TAU compared to TAU only for patients with chronic low back pain and comorbid PTSD. Contrary to what was expected, both groups achieved a moderate reduction in disability and pain. Also, both groups achieved a small reduction in pain catastrophizing. As expected, the SE intervention in addition to TAU significantly reduced the number of PTSD symptoms compared with TAU alone. These results support previous findings indicating that comorbid PTSD is not an obstacle for chronic pain management (e.g. Andersen et al., 2014). Kinesiophobia was significantly reduced, with a moderate effect size in the SE group only. This reduction in kinesiophobia in the SE group is in accordance with the rationale of SE: that an interoceptive approach has a positive effect on experiential avoidance of bodily sensations. A better tolerance or an increase in self-regulatory skills is expected to have a positive effect on kinesiophobia or fear of pain associated with movement. The results support the notion that changes in the fear network can be achieved by mechanisms other than direct exposure to trauma memories (Peri et al., 2015).

Although a moderate reduction in kinesiophobia and PTSD symptoms were achieved for the SE group, the overall effect of SE was less than expected. In particular, the clinical importance of the outcomes can be questioned. For instance, in low back pain, the recommended minimal clinically meaningful improvement in pain and pain-related disability is 30% change from baseline (Ostelo et al., 2008). Our results are in accordance with the CBT trials targeting motor vehicle-related PTSD (Beck et al., 2009; Dunne et al., 2012). With that said, the results are still encouraging when taking into account that the SE intervention was very brief and the trauma symptoms were severely chronic and in many cases related to complex traumatic events such as interpersonal trauma dating back decades. In the current sample, the mean duration with possible PTSD/sub-clinical PTSD was > 10 years, and the majority had experienced more than one traumatic event. Almost, one-third of the index traumas were interpersonal traumas, which may be of a more complex nature compared to traffic injuries. Also, that the effect was present 12-months post treatment indicates that the effect is long lasting. Finally, tolerance to the intervention was high, which is reflected in few dropouts.

One may speculate whether a more intensive SE intervention in combination with TAU could have increased the effect on PTSD and pain. Also, as suggested by the mutual maintenance model (Sharp & Harvey, 2001), if PTSD and comorbid pain is the result of the same traumatic event, pain can trigger intrusive thoughts exacerbating PTSD and vice versa. Hence, a more stratified treatment for patients with comorbid PTSD and pain related to the same traumatic event may increase the effect of SE on both PTSD and pain. Finally, third wave cognitive behavioural therapies fostering acceptance have been found to be promising in addition to traditional CBT approaches for chronic pain (e.g. McCracken & Morley, 2014). For this reason, SE, which in many ways resembles third wave approaches, may be an effective additional treatment in the context of chronic pain rehabilitation. However, the current study indicates that SE in itself is not sufficient for alleviating comorbid PTSD and pain. Also, in the case of more chronic or complex PTSD, interventions may need to be more individually tailored. However, to date, there are still not sufficient data to indicate whether PTSD and pain are affected by the same intervention. Also, it is not known whether the effect of standard pain rehabilitation can be generalized to PTSD, or whether the two conditions need to be targeted separately or simultaneously (Beck & Clapp, 2011).

## 4. Limitations

The current study has several limitations. First, the inclusion of patients with mixed traumas dating back decades is a challenge, making it difficult to interpret whether a stricter inclusion criterion could increase the effect of SE. Since the primary outcome of the study was pain-related disability, one should be cautious when drawing any conclusions about the efficacy of the SE intervention for PTSD. To test the efficacy of SE would require a control group also targeting PTSD symptoms. Also, more follow-up times would have been valuable in the interpretation of potential mediating mechanisms related to the outcomes. Moreover, any future study should include a more manualized control condition. Finally, the prevalence and number of traumatic events experienced should be interpreted with caution, since traumatic events were reported in very broad categories and not on a detailed trauma checklist.

## 5. Conclusion

A brief Somatic Experiencing intervention in addition to treatment as usual was found to have a significant effect on post-traumatic stress disorder and kinesiophobia compared to treatment as usual alone. The results are promising when taking into consideration the complexity of the sample and the effect being present at the 12-month follow-up. However, it still remains inconclusive as to whether more sessions of SE and a more select sample of patients with comorbid post-traumatic stress disorder and pain related to the same event, such as a traffic injury, may increase the effect of SE on pain and disability.

## Highlights of the article

- The current study is the first randomized controlled trial evaluating the effect of Somatic Experiencing (SE) for comorbid PTSD and low back pain.
- A brief SE intervention in addition to TAU significantly reduced PTSD symptoms and fear of movement compared with TAU alone (supervised exercises for low back pain).
- Both groups achieved a large reduction in disability and pain.
- A brief SE intervention is not sufficient to fully alleviate comorbid PTSD and pain.
